# Supplementary material for: NMR-Based Metabolomics Reveals Position-Specific Signatures Associated with Physical Demands in Professional Soccer Players
Source: Biomedicines. 2025 Oct 22;13(11):2583. doi: 10.3390/biomedicines13112583 (PMC12650136; doi:10.3390/biomedicines13112583)
Supplement: Supplementary file 1 [file biomedicines-13-02583-s001.zip › Supplementary material.pdf]

Supplementary material

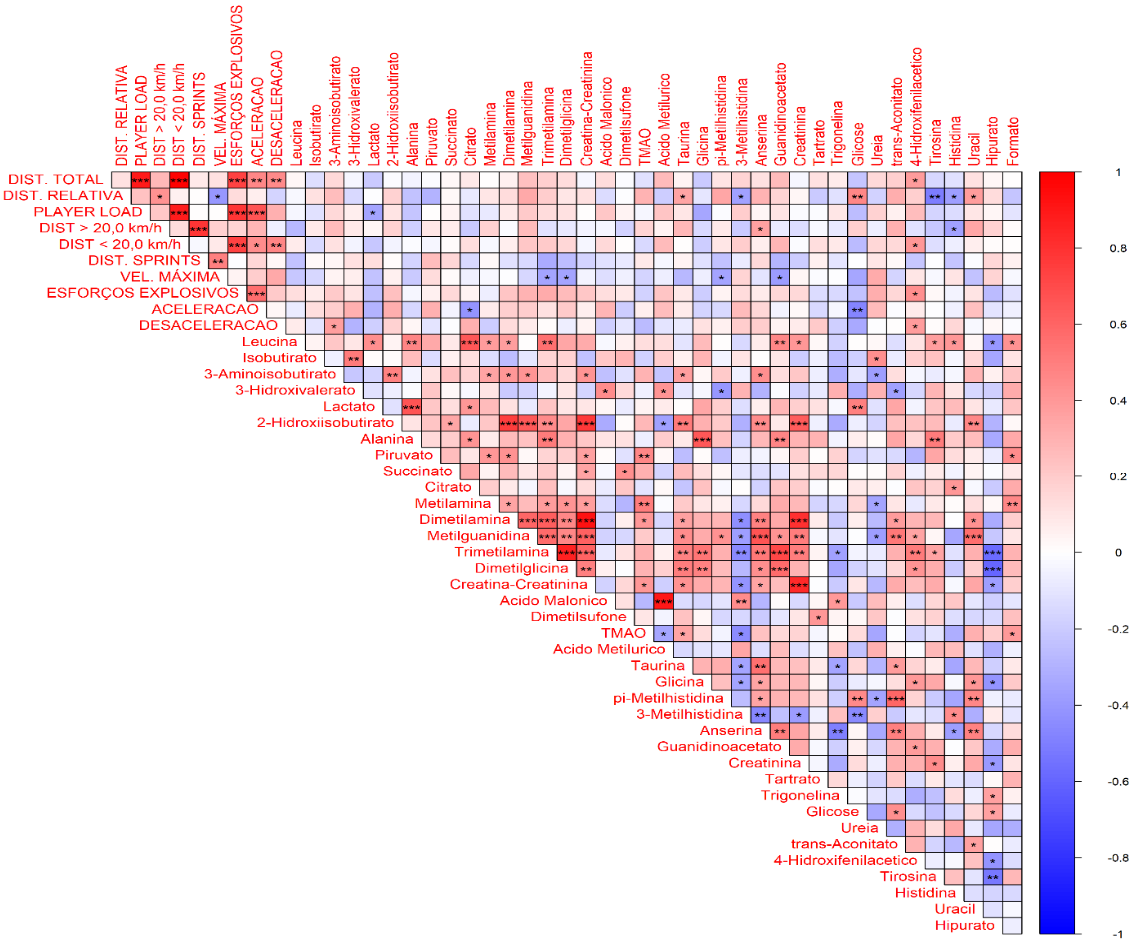

Figure S1. Complete Pearson correlation matrix displaying all pairwise correlations between the 38 urinary metabolites and external load variables following championship matches for the full-back (FB), central midfielder (CM), and central defender (CD) positions. The color scale represents positive (red) and negative (blue) correlations, with significance levels indicated by asterisks: *$p < 0.05$, **$p < 0.01$, ***$p < 0.001$.
